# Effects of physical activity participation on subjective well-being of Chinese residents: mediating effects of physical health status and perceived social development

**DOI:** 10.3389/fpsyg.2025.1415158

**Published:** 2025-01-23

**Authors:** Pingqiang Wei, Ting Qin, Chengyi Zhu

**Affiliations:** ^1^School of Literature and Journalism, Xihua University, Chengdu, China; ^2^School of Computer and Software Engineering, Xihua University, Chengdu, China

**Keywords:** physical activity participation, subjective well-being, physical health status, perceived social development, mediating effect

## Abstract

**Background:**

Physical exercise participation can effectively improve the subjective well-being of Chinese residents; however, further research is needed to understand how this participation influences their well-being. This study aims to explore the influence mechanism of physical exercise participation on the subjective well-being of Chinese residents and the mediating role of physical health status and social development cognition in this relationship.

**Methods:**

Based on the data from China’s comprehensive social survey in 2021, 7,923 valid samples were selected. Three variables, such as physical exercise participation, physical health status, and social development cognition, were used to explore residents’ subjective well-being. Multiple linear regression models, structural equation models, and bootstrap methods were employed to assess the mediating effects of these variables.

**Results:**

(1) Physical exercise participation can improve residents’ subjective well-being ( β =0.084, *t* = 4.67) and promote the development of positive psychology. (2) Physical health status has a mediating effect on the impact of physical exercise participation on residents’ subjective well-being, with a mediating effect calculated at 0.05 (95% CI [0.01, 0.09]). (3) The cognitive level of social development has a mediating effect on the influence of physical exercise participation on residents’ subjective well-being, with a documented mediating effect of 0.04 (95% CI [0.01, 0.08]). (4) Both physical health status and social development cognition collectively exhibit a chain mediating effect on the relationship between physical exercise and subjective well-being, with a combined mediating effect of 0.07 (95% CI [0.04, 0.10]).

**Conclusion:**

Participation in physical activity has a positive effect on residents’ subjective well-being, with physical health status and social development cognition serving as chain mediators in this relationship. Consequently, it is recommended that both government and various societal sectors intensify efforts to promote physical activity. By creating supportive environments that facilitate regular physical exercise, we can enhance the health and happiness of the population at large.

## 1 Introduction

In recent years, the economic architecture and industrial structure of Chinese society have undergone remarkable changes, affecting not only people’s material lives but also their spiritual lives. With the accelerated pace of life and increased competitive pressure, many people have begun to feel unprecedented psychological pressure. These pressures often stem from various sources, such as work, family, and interpersonal relationships, and manifest themselves in mental health problems such as anxiety and depression. These problems not only affect an individual’s daily life and work efficiency but may also have a negative impact on the family and society. Therefore, it is necessary to address the mental health issues among Chinese residents and devise effective strategies to resolve them. Subjective well-being refers to an individual’s overall evaluation of their quality of life, encompassing their satisfaction with life and the experience of positive and negative emotions (Panza et al., 2019; Suh et al., 1998). The level of subjective well-being directly affects an individual’s mental health and plays a crucial role in promoting overall mental well-being (Wren-Lewis and Alexandrova, 2021). Studies have shown that subjective well-being positively impacts residents’ physical and mental health (Yıldırım and Arslan, 2022; Lombardo et al., 2018). It helps individuals cope with life’s pressure and reduces the occurrence of psychological problems. Although scholars have conducted more studies on subjective well-being, there are fewer studies on how physical activity participation affects Chinese residents’ subjective well-being. Therefore, based on previous studies, it is important to further validate how physical activity participation affects Chinese residents’ subjective well-being, thereby contributing to deepening research in the field of subjective well-being and health.

It has been gradually recognized that participation in physical activity plays an important role in maintaining and enhancing one’s physical and mental health. Studies have shown that engaging in physical activity positively affects residents’ health and overall quality of life (Kovalenko et al., 2020; Chen, 2024; Guo and Meng, 2020). Physical activity can improve physical fitness, enhance immunity, and prevent diseases (Wang et al., 2020; Iwon et al., 2021; Shen, 2023). Some studies have shown that regular physical exercise effectively improves the subjective well-being of college students (Yang et al., 2024). Taijiquan, a form of exercise, can positively impact the subjective well-being of the elderly (Wang et al., 2023). These findings suggest that various groups of people can improve their subjective well-being through different ways of physical exercise. In addition, physical activity can reduce psychological problems, such as anxiety and depression, and improve mental health (Panza et al., 2019; Rodrigues et al., 2022; Tian et al., 2022). Therefore, physical exercise has become one of the most important methods for enhancing public health and improving the overall quality of life. Engaging in physical activities can help individuals meet new friends and expand their social circles. However, it may also lead to unpleasant experiences that affect subjective well-being, particularly due to differing ideas or competitive pressures among participants.

Physical health status is the basis for people’s pursuit of a high quality of life and impacts their overall well-being and quality of life. Good physical health is physically and mentally pleasurable and ensures freedom of movement in daily life and freedom from illness. Studies have shown that physical exercise can promote physical health, improve an individual’s fitness level and immunity, and reduce the occurrence of physical diseases (Pu et al., 2023; Ge et al., 2023). Therefore, we predicted residents’ subjective well-being through the mediating role of physical health status.

Social development cognition reflects people’s perception of the social environment and their expectations and attitudes toward future social development. Positive social development cognition can see the prosperity and progress of the society and have confidence in the society and the future (Kim et al., 2021; Ma et al., 2022; Sun et al., 2023). Evidence shows that women’s social and personal cognition positively and negatively affect subjective well-being (Qin et al., 2024). Therefore, negative perceptions of social development, marked by confusion and concern about society and the future, can diminish personal subjective well-being. Through physical activity participation, people can gain more opportunities for social engagement, learn about social information, and develop social relationships and emotional support networks (Won et al., 2020; Zhang et al., 2022). These enhancements in cognitive abilities for social development may directly or indirectly affect residents’ subjective well-being.

There is an interactive relationship between physical health status and social development cognition. On the one hand, good physical health provides individuals with more opportunities and energy to participate in social activities, which in turn enhances their social development perceptions (Zhaoyang et al., 2019; Wang et al., 2019); on the other hand, greater perceptions of social development motivate individuals to pay more attention to their physical health, leading to increased participation in physical activities more actively (Huang et al., 2024). However, there is no direct evidence of the relationship between physical activity participation and physical health status, social development cognition, and subjective well-being of Chinese residents.

In summary, there is a close relationship between physical activity participation, physical health status, social development cognition, and Chinese residents’ subjective well-being. Although related studies have revealed the relationships among the four variables separately, few studies have constructed chain mediation models around these four variables. Constructing a chain-mediated model of the relationship between physical activity participation, physical health status, social development perceptions, and subjective well-being is an important perspective for exploring the path of individual well-being enhancement in depth. This model not only helps us understand how physical activity participation affects residents’ subjective well-being through multiple pathways but also provides a scientific basis for policymakers and the community to promote health and social well-being for all. Based on this understanding, this study proposes the following hypotheses:

*Hypothesis 1*: A positive relationship exists between physical exercise participation and residents’ subjective well-being.

*Hypothesis 2*: Physical health plays an intermediary role between physical exercise participation and residents’ subjective well-being.

*Hypothesis 3*: Social development cognition plays an intermediary role between physical exercise participation and residents’ subjective well-being.

*Hypothesis 4*: Physical health status and social development cognition has a chain mediating effect in the process of physical exercise participation, affecting residents’ subjective well-being.

## 2 Research methods

### 2.1 Data sources

The data for this study are sourced from China’s General Social Survey (CGSS). To ensure the timeliness and accuracy of the findings, we selected the latest data released by CGSS in 2021 [hereinafter referred to as CGSS] for analysis. The initial sample size of CGSS (2021) data is 8,148, covering 28 provinces, autonomous regions, and municipalities in China (excluding Xinjiang, Tibet, Hainan, Hong Kong, Macao, and Taiwan). CGSS data have national, comprehensive, continuous, and modular characteristics and are highly representative of Chinese society. The CGSS data sampling method is scientific, of high quality, and wide-ranging, which provides valuable micro-data for both domestic and foreign researchers who want to study Chinese families and social problems. The conclusions obtained by using the CGSS data set are universal and accurate for the study of the Chinese population society, which is very suitable for this study. For this study, given its specific needs and objectives, a meticulous screening and preprocessing of the raw data was conducted. This process was aimed at eliminating samples that were unrelated to the study, as well as removing incomplete data or obvious errors, to ensure the accuracy and reliability of the final analysis results. After this thorough elimination and cleaning work, we successfully obtained 7,923 high-quality sample data points that met the requirements of the research.

### 2.2 Variables measurement

#### 2.2.1 Dependent variable

The subjective well-being of Chinese residents was selected as the dependent variable in this study. Khalek et al. assessed well-being by asking, “Are you generally happy?” (Abdel-Khalek, 2011). Meanwhile, Shi et al. selected the CGSS “Do you feel happy in your life?” question as a subjective well-being variable (Shi et al., 2022; Zhang et al., 2024; Jin et al., 2020). Using a single question to measure subjective well-being is credible and reliable. For this reason, the question “In general, do you feel that you are happy in your life?” from the CGSS (2021) was selected for this study. This question was selected as the dependent variable for this study. The value of “very unhappy” is assigned as 1, “relatively unhappy” as 2, “cannot say if I am happy or not” as 3, “relatively happy” as 4, and “very happy” as 5. The larger the value, the stronger the subjective happiness of the residents.

#### 2.2.2 Independent variables

In this study, physical exercise participation is selected as the independent variable. The question from the China General Social Survey (CGSS) 2021, “In the past year, do you often participate in physical exercise in your free time?” is used to measure this variable. Responses indicating frequent exercise participation, such as “every day,” “several times a week,” and “several times a month,” as well as “several times a year or less,” are assigned a value of 1, indicating active participation in physical exercise. Conversely, the response “never” is assigned a value of 0, indicating no participation in physical exercise. This coding approach categorizes and quantifies the level of physical activity among the study’s participants.

#### 2.2.3 Mediator variables

Physical health status and social development cognition were selected as the mediating variables in this study. The physical health status variable was selected, and the question “I am very distressed about my health status” in CGSS (2021) was selected. The “very disagree” option was assigned to 5, which represents very healthy; the option “disagree” is assigned to 4, which represents health; the options “somewhat disagree” and “somewhat agree” are combined and assigned a value of 3, representing a general health condition; the option “agree” is assigned to 2, which means unhealthy; assigning the option “agree very much” to 1 represents very unhealthy. The cognitive variable of social development is selected, and the question “I am very confident in the development of society” in CGSS (2021) is selected. The option “very disagree” is assigned to 1, which represents very little confidence; the option “disagree” is assigned to 2, which means no confidence; it combines the options “a little disagree” and “a little agree” with a value of 3, which represents the general; the option “agree” is assigned to 4, which means confidence; by assigning the option “agree very much” to 5, it means that you are very confident.

#### 2.2.4 Control variables

The social demographic characteristics are selected as the control variables in this study. Sociodemographic variables include gender, ethnicity, religious beliefs, education level, and socioeconomic status. For gender variables, this study assigned “male” to 1 and “female” to 2. For ethnic variables, this study assigned “Han” to 1 and “ethnic minorities” to 2 (the remaining 55 ethnic groups combined to 2). For education level variable, the question “Your current highest education level?” was selected. The option “no education” is assigned to 1; the option of “private school, literacy class” is assigned to 2; the option “primary school” is assigned to 3; the option “junior high school” is assigned to 4; the option “vocational high school” is assigned to 5; the option “ordinary high school” is assigned to 6; the option “technical secondary school” is assigned to 7; the option “technical school” is assigned to 8; the option “university college (adult higher education)” is assigned to 9; the option “college (formal higher education)” is assigned to 10; the option “undergraduate (adult higher education)” is assigned to 11; the option “undergraduate (formal higher education)” is assigned to 12; and the option “graduate and above” is assigned to 13. The larger the value, the higher the education level. For socioeconomic status variables, the question, “Overall, in the current society, your own socioeconomic status belongs to?” was selected. The option “lower layer” is assigned to 1; “middle and lower” to 2; “middle” to 3; “upper middle” to 4; and “upper” to 5. The larger the value, the higher the socioeconomic status.

### 2.3 Data analysis method

#### 2.3.1 Multiple linear regression model

The mathematical equation of the multivariate linear regression model in this study can be expressed as Equation 1:


(1) $$Y=\beta_{0}+\beta_{1}X_{1}+\beta_{2}X_{2}+\beta_{3}X_{3}+\beta_{4}X_{4}+\in$$

In the Equation 1, Y is the subjective well-being of residents, that is, the model’s dependent variable. $X_{1}$ is the degree of participation in education and exercise, which is the main independent variable concerned in this study. $X_{2}$ , $X_{3\text{,}}$ and $X_{4}$ This study’s control variables are socio-demographic characteristics, physical health status, and social development cognition. $\beta_{0}$ is the intercept term, which indicates the expected value of the dependent variable when all the independent variables are 0. $\beta_{1},\beta_{2},\beta_{3},\beta_{4}$ are regression coefficients, which represent the degree of influence of each variable on the dependent variable. Among them, $\beta_{1}$ is the coefficient of special concern in this study, which indicates the impact of physical exercise participation on subjective well-being. ∈ is the error term.

#### 2.3.2 Structural equation model

The mathematical equation of the structural equation model in this study can be expressed as Equation 2:


(2) Y=By+Γx+ζ

In the Equation 2, y is the independent variable vector, x is the other variable vector, B is the path coefficient matrix between independent variables, Γ is the path coefficient matrix between other variables and independent variables, and ζ is the residual term of the structural equation model. Through the constructed structural equation model, the bootstrap method is used to estimate the mediating effect and test its significant effect on the chain mediating effect.

This model uses χ^2^ /df, RMR, RMSEA, NFI, RMAE, and other fitness test indexes to fit the model. The specific model index fitting standard is shown in Table 1.

**Table 1:** Model fitting degree table.

Index	Fit index	Standard
Absolute index	χ^2^ /df	<3
	RMR	<0.05
	GFI	>0.9
	RMSEA	<0.1
Value-added indicators	NFI	>0.9
	CFI	>0.9
	NNFI	>0.9
Residual index	RMAE	>0
	R^2^	<1

## 3 Results

### 3.1 Study the statistical results of variables

**Figure 1:**
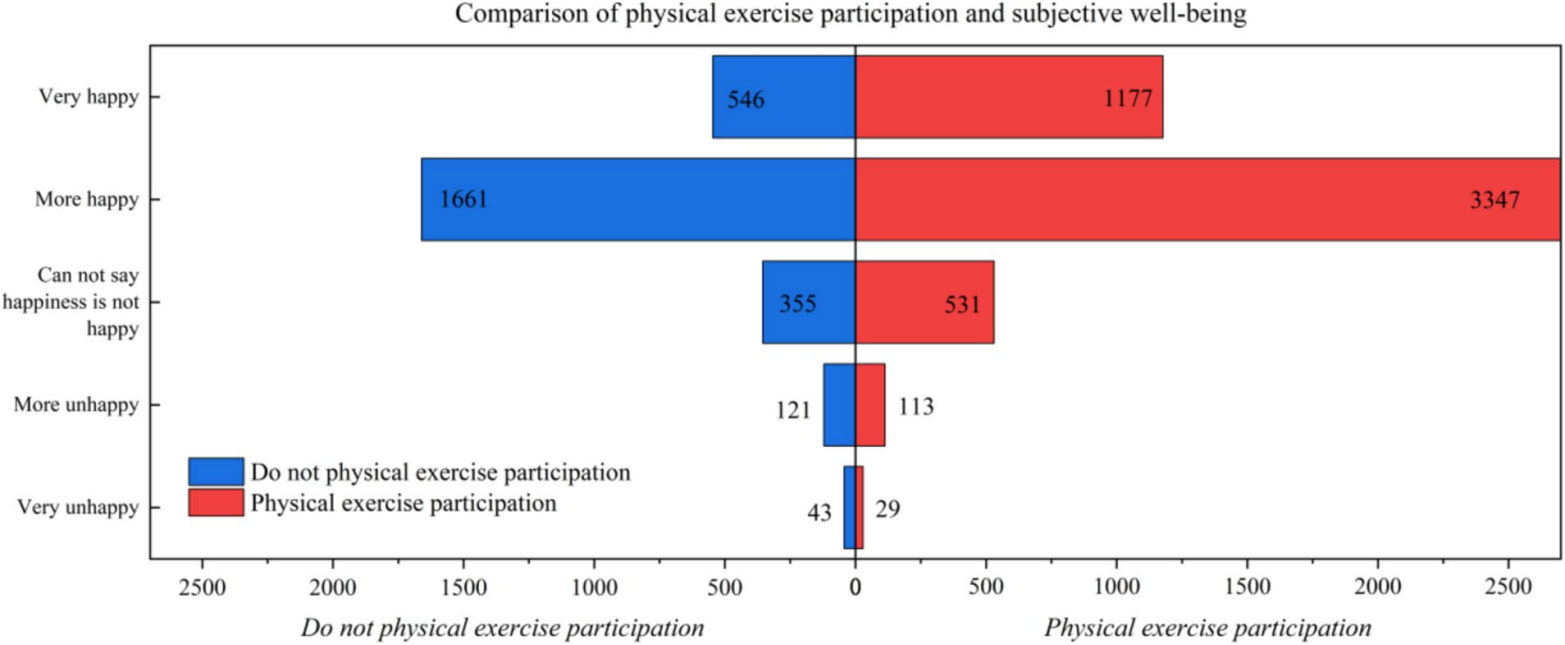
Comparison of physical exercise participation and subjective well-being.

According to statistics, residents who engage in physical exercise report an average 11.3% increase in subjective well-being compared to those who do not, as shown in Figure 1.

The variables of this study were statistically analyzed, and the descriptive statistical results of each variable are shown in Table 2.

**Table 2:** Descriptive result statistics of each variable.

	*M*	*SD*	*Min*	*Max*	*N*
Sexuality	1.55	0.498	1	2	7,923
Nation	1.05	0.212	1	2	7,923
Education degree	5.35	3.330	1	13	7,923
Socioeconomic status	3.68	0.930	1	5	7,923
Subjective well-being	4.02	0.727	1	5	7,923
Physical exercise	0.66	0.475	0	1	7,923
Health status	3.35	0.909	1	5	7,923
Social development cognition	3.98	0.691	1	5	7,923

It can be seen from Table 2 that the sample data of each variable are 7,923. The score of subjective well-being (dependent variable) ranged from 1 to 5, with a mean of 4.02 (standard deviation SD = 0.727), indicating that most samples had higher subjective well-being. The mean value of physical exercise participation (main independent variable) is 0.66 (standard deviation *SD* = 0.475), the minimum value is 0 (“no physical exercise”), and the maximum value is 1 (“physical exercise”), which shows that Chinese residents have a higher frequency of physical exercise participation.

### 3.2 Multiple regression results

In this study, the subjective well-being of Chinese residents was selected as the dependent variable, the participation in physical exercise was selected as the independent variable, and the social demographic characteristics, physical health status, and cognitive level of social development were selected as the control variables of the research model. To establish the multiple linear regression model of this study, the model results are shown in Table 3. The numerical values in brackets in Table 3 represent the standard error coefficient, and the data results shown in Table 3 are within the confidence interval of the model. The significant influence coefficient *P* is expressed by *, and the influence coefficient *β* between model variables is expressed by numerical values outside brackets. Only socio-demographic characteristics are selected in Model 1. Model 2 adds physical health factors based on Model 1. Model 3 adds social development cognitive level factors based on Model 2. Model 4 includes all control variables and independent variables. According to Models 5 and 6, the Chinese residents are divided into two groups—men and women—based on gender, and the regression analysis is carried out to further explore whether the gender difference between men and women will have an impact on the results.

**Table 3:** Multiple linear regression results of influencing factors of residents’ subjective well-being.

	1	2	3	4	5	6
Sexuality	−0.023**(0.016)	−0.022**(0.016)	−0.022**(0.016)	−0.021**(0.016)	—	—
Nation	0.007*(0.038)	0.008*(0.038)	0.010*(0.038)	0.008*(0.038)	−0.015(0.057)	0.023(0.051)
Education degree	0.008*(0.002)	0.008**(0.002)	0.008*(0.002)	0.004*(0.003)	0.004(0.004)	0.004(0.004)
Socioeconomic status	−0.139**(0.009)	−0.139**(0.008)	−0.138**(0.009)	−0.134**(0.009)	−0.151(0.013)	−0.120(0.012)
Health status	—	0.065**(0.009)	0.062**(0.009)	0.060**(0.009)	0.053**(0.013)	0.064**(0.012)
Social development cognition	—	—	0.441**(0.012)	0.440**(0.012)	0.423**(0.017)	0.410**(0.016)
Exercise participation	—	—	—	0.084**(0.018)	0.057(0.027)	0.105**(0.025)
*N*	7,923	7,923	7,923	7,923	3,581	4,342
Constant term	4.512**(0.061)	4.475**(0.069)	4.413**(0.084)	4.364**(0.084)	4.442**(0.119)	4.251(0.107)
Adjusted R^2^	0.502	0.579	0.598	0.605	0.443	0.545

*p < 0.05, **p < 0.01, standard error in parentheses.

The study found that the regression coefficient for physical exercise participation is *β* = 0.084 (*p* = 0.000 < 0.01), which indicates that physical exercise participation has a significant positive impact on subjective well-being, thus confirming Hypothesis 1. In Model 4, when all control and independent variables are included, the fitting effect of Model 4 is improved compared with Model 3, indicating that the model established in this study is better. According to the results of Model 4, under the influence of control variables, participation in physical exercise still positively impacts the subjective well-being of Chinese residents. Compared with residents who do not engage in physical exercise, subjective well-being increases by 0.084 points on average, indicating that residents who participate in physical exercise are more likely to feel more subjective well-being.

According to the results of multiple linear regression, the regression coefficient of physical health status is *β* = 0.065 (*p* = 0.000 < 0.01), and the regression coefficient of social development cognition is *β* = 0.441 (*p* = 0.000 < 0.01). It means that both physical health status and social development cognitive level will have a significant positive impact on subjective well-being. Therefore, residents with good physical health and confidence in social development have stronger subjective well-being.

According to the results of the overall sample model, gender factors still have an impact on residents’ subjective well-being after other variables are controlled. Specifically, women who regularly exercise will score 0.105 points higher in subjective well-being than women who do not exercise; for men, it is not significant. This shows that men and women, through participation in physical exercise to improve the degree of subjective well-being, are different. The differences may be between men’s and women’s social development, cognitive level, physical health status, and other factors that influence their subjective well-being.

### 3.3 Structural equation model

According to the structural equation model established in this study, the calculated model parameters are shown in Table 4.

**Table 4:** Model fitting degree table.

Index	Fit index	Actual value	Result
Absolute index	χ^2^ /df	1.345	Excellent
	RMR	0.032	Excellent
	GFI	0.963	Excellent
	RMSEA	0.036	Excellent
Value-added indicators	NFI	0.974	Excellent
	CFI	0.975	Excellent
	NNFI	0.978	Excellent
Residual index	RMAE	0.574	Excellent
	R^2^	0.735	Excellent

It can be seen from Table 4 that the actual values of the above model fitting indexes are all within the range of judgment criteria, indicating that the fitting degree of this model is very good. Therefore, it is considered that the structural equation model has a good fitting effect on the data of this study. The results of this model are shown in Table 5.

**Table 5:** Results of the structural equation model.

Latent variable	→	Manifest variables	Standardized coefficient	*P*
Exercise participation	→	Social development cognition	0.043	0.000***
Social development cognition	→	Subjective well-being	0.044	0.002***
Exercise participation	→	Subjective well-being	0.045	0.002***
Health status	→	Subjective well-being	0.079	0.000***
Socio-demographic characteristics	→	Subjective well-being	0.034	0.000***
Health status	→	Social development cognition	0.039	0.000***
Exercise participation	→	Health status	0.059	0.001***

***, **, * represents the significance levels of 1, 5, and 10%, respectively.

**Figure 2:**
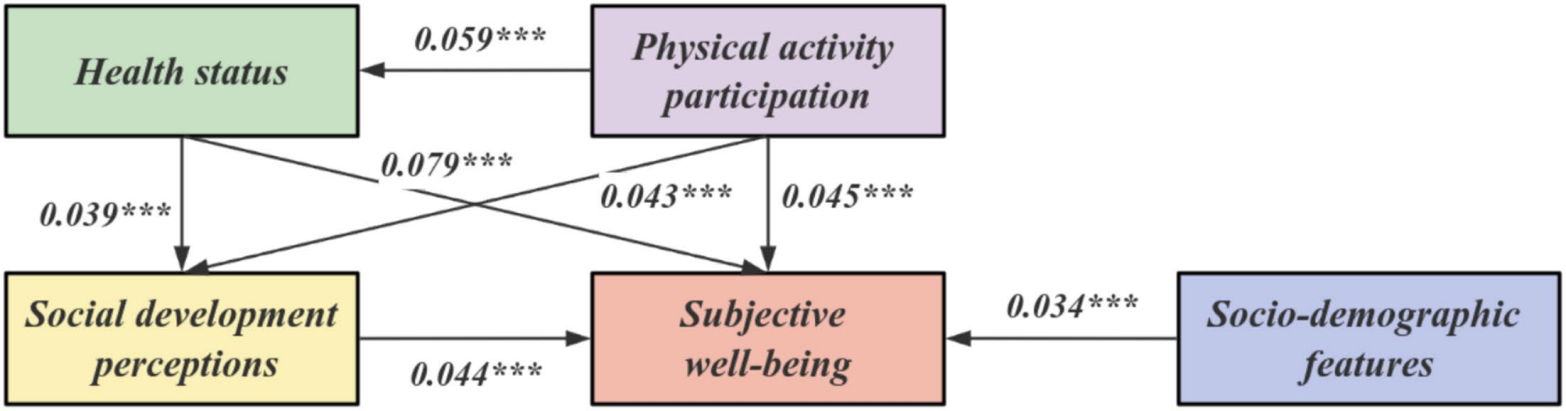
Path result diagram of structural equation model.

According to the model results, the significant *p*-values of the measured variables of each variable are less than 0.05, and the standardized load coefficient values of each variable are also greater than 0.4. Therefore, it can be shown that the measured variables in this model meet the requirements of the structural equation model factors. It can be considered that it has a sufficient variance interpretation rate to show that each variable can be shown on the same factor. It can be seen from Table 5 that the latent variable of this model→explicit variable: physical exercise participation→social development cognition, path influence coefficient *β* = 0.043. Social development cognition→subjective well-being, path influence coefficient *β* = 0.044. Physical exercise participation→subjective well-being, path influence coefficient *β* = 0.045. Physical health→subjective well-being, path influence coefficient *β* = 0.079. Social demographic characteristics→subjective well-being, path influence coefficient *β* = 0.034. Physical exercise participation→physical health status, path influence coefficient *β* = 0.039. Physical health status→social development cognition, path influence coefficient *β* = 0.059. In terms of the above paths, the significance *p* values of all pairs of variables are all<0.05, which is significant at the level, so all the paths set up in this model are effective. The path coefficient diagram is shown in Figure 2.

### 3.4 Mediation model results

In this study, the Bootstrap method was used to test the mediating effect, and a mediation model was developed with physical activity participation as the independent variable, subjective well-being as the dependent variable, and physical health status and social development perceptions as the mediating variables. The results of the mediating effect of bootstrap analysis are shown in Table 6.

**Table 6:** Bootstrap analysis of mediating effect.

Category	Effect size	SE	95%CI	Relative mediating effect (%)
The total indirect effect of physical exercise participation and subjective well-being	0.16	0.03	[0.10, 0.22]	61.54
Physical exercise participation→physical health→subjective well-being	0.05	0.02	[0.01, 0.09]	19.23
Physical exercise participation→social development cognition→subjective well-being	0.04	0.01	[0.01, 0.08]	15.92
Physical exercise participation→health status→social cognition→subjective well-being	0.07	0.02	[0.04, 0.10]	26.39

The results in Table 6 show that physical health status and social development cognitive level have a mediating role in the influence of physical activity participation on the subjective well-being of Chinese residents, and the mediating effect is significant. Physical activity participation improves residents’ subjective well-being by improving their physical health status, with a mediating effect of 0.05 and a 95% CI of [0.01, 0.09], and the interval does not include the number 0 for this path, indicating that this mediating effect path exists, thus confirming Hypothesis 2. Moreover, physical activity participation indirectly affects residents’ subjective well-being by influencing their perceived level of social development, with a mediating effect size of 0.04, 95% CI of [0.01, 0.08], and the interval for this path does not include the number 0, which indicates that this mediating effect path exists, thus confirming Hypothesis 3. Finally, the chain mediation effect path is analyzed. For the mediation path of “participation in physical exercise→health status→social cognition→subjective well-being,” physical exercise affects the residents’ social development cognitive level by influencing the residents’ physical health status and finally affects the residents’ subjective well-being. The mediation effect size is 0.07, with a 95% confidence interval (CI) ranging from 0.04 to 0.10. Since this interval does not include the number 0, it confirms the presence of the chain mediation effect. This result confirms Hypothesis 4, affirming that the specified mediation pathway exists within the study.

## 4 Discussion

### 4.1 Influence of physical activity participation on residents’ subjective well-being

This study’s results indicate a positive relationship between physical activity participation and residents’ subjective well-being. This finding is consistent with the results of most existing studies in the current academic community that physical activity helps improve physical health (Tao et al., 2022; Zhang et al., 2022) and promotes social interaction and psychological health, leading to individuals feeling more subjective well-being (Gan and Jiang, 2022; Lin et al., 2022). Participation in physical activity offers numerous benefits, including promoting physical health, improving mental well-being, and fostering social interactions (Suzuki et al., 2020; Zhang et al., 2021). By participating in physical activity, individuals can release stress, improve self-confidence, and enhance self-awareness, thus becoming more active and happy in their daily lives (Cho et al., 2023; Shang et al., 2021; Wendtlandt and Wicker, 2021). However, it is important to note that not all forms of physical activity positively affect subjective well-being, as individual preferences, motivation, and level of participation may affect the effects of physical activity.

### 4.2 Differences in subjective well-being of different groups

From a gender perspective, female individuals show more significant characteristics in the relationship between physical activity participation and subjective well-being (Huang and Zhang, 2022). This may be because females are more involved in physical activity or because they have more positive perceptions of the physical health and social interactions that physical activity provides. In contrast, men may be influenced by more social roles and family responsibilities, leading them to show some differences in the relationship between physical activity participation and subjective well-being (Jiang et al., 2021; Zhang et al., 2021; Zhou and Zhou, 2022). In terms of education, individuals with higher education levels showed more significant positive associations in the relationship between physical activity participation and subjective well-being (Zhang and Wu, 2024). This may be because individuals with higher levels of education are more health-conscious and more able to realize the importance of physical activity for physical health and mental health and thus participate more actively in physical activity activities and consequently achieve higher subjective well-being.

### 4.3 Mediating role of physical health level

Physical health is considered an important component of subjective well-being, and physical exercise, as an activity that promotes physical health, indirectly enhances an individual’s subjective well-being by increasing his or her level of physical health (Martín-María et al., 2020; Chen et al., 2022). Improved health status can directly reduce psychological stress and negative emotions such as anxiety and depression, thus enhancing emotional stability and positivity. Healthy individuals are more inclined to engage in social activities, and adopting a healthy lifestyle fosters a positive outlook on life, encouraging comprehensive and balanced personal development and enhancing their overall sense of well-being. However, it should be noted that physical health not only refers to the health status of the body but also includes the perception of and attitude towards one’s physical condition. Therefore, in addition to objective physiological indicators, an individual’s subjective perception of his or her health is also an important factor affecting subjective well-being (Mills et al., 2019; Wang et al., 2021).

### 4.4 Mediating role of social cognitive level

Enhanced social cognition allows individuals to better comprehend their social environment and position, fostering positive relationships and emotional connections, which, in turn, improve subjective well-being. However, the improvement of social cognitive level may be affected by various factors, including but not limited to educational attainment, socioeconomic status, and cultural background (Zhang and Li, 2021; Zhang et al., 2023; He and Wang, 2023). In addition, the mediating effect of the level of social cognition between physical activity and subjective well-being may pose some problems. Excessive social developmental cognitions may lead to overly high expectations of physical activity. When the effects of physical activity fail to meet expectations, individuals may feel disappointed and frustrated, which in turn reduces subjective well-being. Even if people recognize the importance of physical activity, they may not be able to adhere to it because of factors such as time and energy. This discrepancy between perception and actual action may also negatively affect subjective well-being.

### 4.5 Chain mediating effects of physical fitness levels and social cognitive levels

The study shows that physical health level and social cognitive level have a chain mediating effect in the process of physical activity participation in influencing residents’ subjective well-being. This process can be understood as a gradual and interactive process: the improvement of physical health makes individuals more capable of participating in social activities and actively exploring the outside world, thus enhancing their social cognition, and the improvement of social cognition further strengthens the individual’s attention to and protection of physical health, thus forming a virtuous circle. Physical health can not only directly affect an individual’s quality of life and subjective well-being but also indirectly affect his or her social cognitive level by influencing the individual’s perception and cognition of the social environment (García-Hermoso et al., 2020; Yuan and You, 2022). For example, an individual in good physical health may be more capable of engaging in social activities and actively exploring the outside world, thus having a richer social experience and cognition. Conversely, an individual with poor physical health may be more likely to feel tired and passive, and his or her perceptions and understanding of the external world may be compromised (Bae, 2022; Bae and Kang, 2022).

## 5 Conclusion

This study explores the impact of physical exercise participation on the subjective well-being of Chinese residents through the chain mediation model and the mediating role of physical health status and social development cognition. The following conclusions are drawn: (1) Physical exercise participation, physical health status, and social development cognition can positively affect residents’ subjective well-being. Residents who participate in physical exercise have an average increase of 11.3% in subjective well-being compared to those who do not participate in physical exercise. Girls who often participate in physical exercise are 0.105 points higher in subjective well-being than girls who do not participate in physical exercise, but this is not significant for men. (2) Physical exercise participation can directly affect residents’ subjective well-being and indirectly affect residents’ subjective well-being through the mediating role of physical health status and social development cognition. Physical exercise participation affects residents’ social development cognition by affecting residents’ physical health status and finally affects residents’ subjective well-being. In short, this study provides Chinese empirical evidence for the impact of physical exercise participation on subjective well-being and has important guiding significance and practical application value for promoting residents to participate in physical exercise activities and improving their subjective well-being and quality of life.

## 6 Research limitations and future research directions

This study discusses the influence mechanism of physical exercise participation on the subjective well-being of Chinese residents, especially focusing on the mediating role of physical health status and social development cognition in this process. According to the research results, we propose that residents can gradually improve their subjective well-being and enjoy a healthy and happy life by formulating a fixed exercise plan, choosing a favorite way of exercise, participating in sports communities or groups, paying attention to physical health, and promoting social development cognition. In particular, governments and various societal sectors should actively promote physical exercise, enhance public understanding of healthy lifestyles, encourage participation in sports, and foster positive perceptions of social development.

However, this study still has some limitations. First, this study is based on the cross-sectional data of CGSS (2021), which limits our ability to directly infer causality. Cross-sectional design cannot capture the dynamic progression of variables over time. Therefore, although we have found the association between physical exercise, physical health, social development cognition, and subjective well-being, we cannot accurately determine the causal relationship between these variables and their timing. Future research can use a longitudinal tracking design. By collecting data regularly, we can observe the trend of variables such as physical exercise habits, physical health status, social development cognition, and subjective well-being over time and explore the interaction mechanism between them. Second, the self-selection bias in the sample may affect the universality of the results because the individuals participating in the survey may be different from the overall population in terms of physical exercise habits, health status, or subjective well-being. Future research could focus on subgroup analyses to explore the diverse impacts of physical exercise on subjective well-being across different populations. Finally, the measurement of variables in this study depends on the self-report of respondents, which may lead to a certain degree of measurement error. Future research can build a comprehensive model with more relevant variables. These variables can include psychological resilience, social support networks, economic status, education level, environmental factors, and so on. By integrating these variables, we can reveal the complex path and multiple mediating effects of physical exercise on subjective well-being.

## Data Availability

The original contributions presented in the study are included in the article/supplementary material. Further inquiries can be directed to the corresponding author.
